# A Method for Detecting Positive Growth Autocorrelation without Marking Individuals

**DOI:** 10.1371/journal.pone.0076389

**Published:** 2013-10-28

**Authors:** Mollie E. Brooks, Michael W. McCoy, Benjamin M. Bolker

**Affiliations:** Department of Biology, University of Florida, Gainesville, Florida, United States of America; University of Westminster, United Kingdom

## Abstract

In most ecological studies, within-group variation is a nuisance that obscures patterns of interest and reduces statistical power. However, patterns of within-group variability often contain information about ecological processes. In particular, such patterns can be used to detect *positive growth autocorrelation* (consistent variation in growth rates among individuals in a cohort across time), even in samples of unmarked individuals. Previous methods for detecting autocorrelated growth required data from marked individuals. We propose a method that requires only estimates of within-cohort variance through time, using maximum likelihood methods to obtain point estimates and confidence intervals of the correlation parameter. We test our method on simulated data sets and determine the loss in statistical power due to the inability to identify individuals. We show how to accommodate nonlinear growth trajectories and test the effects of size-dependent mortality on our method's accuracy. The method can detect significant growth autocorrelation at moderate levels of autocorrelation with moderate-sized cohorts (for example, statistical power of 80% to detect growth autocorrelation *ρ* ^2^ = 0.5 in a cohort of 100 individuals measured on 16 occasions). We present a case study of growth in the red-eyed tree frog. Better quantification of the processes driving size variation will help ecologists improve predictions of population dynamics. This work will help researchers to detect growth autocorrelation in cases where marking is logistically infeasible or causes unacceptable decreases in the fitness of marked individuals.

## Introduction

Ecologists and evolutionary biologists have long been interested in growth in body size. Studies of growth typically focus on differences among means of populations or treatment groups, striving for low variability around the mean to increase statistical power; variation within groups is often treated as noise obscuring the phenomena of interest. However, ecological studies are increasingly considering among-individual variation as either a treatment or a response variable [1]–[6]. These studies have shown that variation among individuals is itself the result of important biological processes and that population dynamics are sensitive to among-individual variation [7]–[12]. These studies have also highlighted challenges for quantifying and explaining the mechanisms underlying observed variation among individuals in a population or cohort [1], [3]. Here, we present a new method that allows the separation of among- and within-individual variation in growth rates based on data from individuals within cohorts measured several times over the course of their ontogeny. In contrast to existing methods, individual organisms need not be marked or otherwise identifiable. By expanding the range of organisms and experimental designs where among-individual variation can be estimated, this method will enable researchers to better understand sources of variation in body size data.

Many ecological and evolutionary processes depend on body size [13]–[17]. Because the body size at which individuals undergo life history transitions is correlated with fitness [18], one branch of life history theory has focused on predicting the size and timing of these transitions [19]–[23]. Among-individual variation in growth rates, largely neglected in this field, can modulate the expected patterns. For example, individuals are known to face a tradeoff between the risk of predation incurred by an aggressive foraging strategy and the risk of desiccation when temporary ponds dry, incurred by the slow growth and development due to a more conservative foraging strategy [24]. The optimal strategy may vary across individuals depending on their genotype and previous foraging success, with fast-growing individuals opting for a riskier strategy. Such growth-mortality risk tradeoffs can lead to flat or bimodal fitness curves that maintain variation in populations [25].

Among-individual variation also modifies population and community dynamics [10], [11], [26], [27]. For example, among-individual variation in developmental rates changes the amplitude and periodicity of population cycles in host-parasitoid models [28]. In population viability analyses, neglecting among-individual variation in survival probability leads to overestimation of extinction risk [29] and underestimation of the population's asymptotic growth rate [30], while neglecting variance in fecundity among individuals may either over- or underestimate the population growth rate [29]. Because survival probabilities and fecundity rates are closely linked to individual body size, variation in individual growth rate may drive changes in demography. Thus, incorporating growth autocorrelation in models may allow more accurate predictions of population size structure [8].

**Figure 1:**
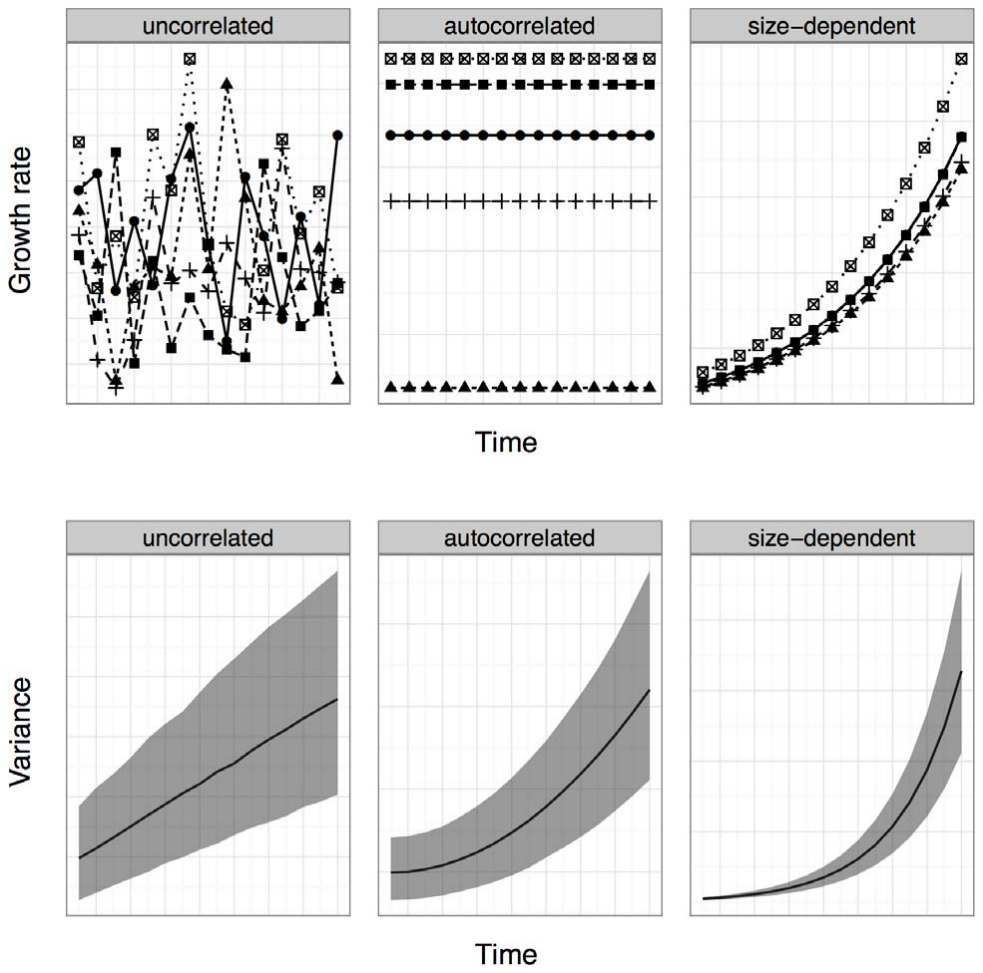
Simulated growth of individuals: growth parameters are the same across panels except for the assumptions: (left) uncorrelated variation among individuals, (center) autocorrelated variation, (right) positively size-dependent growth. A - Patterns of growth rate Growth rates of five individuals are represented in each graph by five separate lines. B - Patterns of size variation Average cohort variance in 2000 cohorts of 50 indviduals: mean (solid line) and 2.5% and 97.5% quantiles (grey ribbons).

Three main growth processes lead to growth depensation (increasing size variation within a cohort through time): within-individual variation in growth rate, among-individual variation in growth rate (i.e., positive growth autocorrelation), and size-dependent growth (Figure 1A) [1].

- Within-individual variation in growth rate occurs when environmental heterogeneity causes uncorrelated temporal variation in individuals' growth rates through time. Here, we take the pattern of growth depensation caused by within-individual variation to be the null expectation.
- Among-individual variation in growth rate, or positive growth autocorrelation, is defined as positive temporal correlation in the growth rate of individuals. Many ecological processes can generate positive growth autocorrelation. The proactive and reactive behavior types discussed above generate permanent autocorrelation (autocorrelation that applies throughout the entire life stage), as individuals consistently express the same behavior pattern [31], [32]. In tree populations, variation in liana load generates permanent autocorrelation [27], while growth autocorrelation driven by extra light availability near treefall gaps is temporary, acting only until an individual near the gap grows up to fill it. In this paper, we focus on growth autocorrelation that persists throughout the time period of interest, although our methods could in principle be adapted to detect temporary autocorrelation.
- Size-dependent growth, where larger individuals have higher expected growth rates, can result from size-dependent gape limitation or size-dependent range size in animals. In plants, size-dependent growth often results from size-dependent resource uptake and asymmetric competition [13]. While size-dependent growth is important, and has frequently been suggested as a mechanism of positive growth autocorrelation [4], it is not our main focus here, although we do discuss below how to control for size-dependent variation when using our method.

These three classes of mechanisms lead to different patterns of variation among individuals in a cohort through time (Figure 1B). Because size-dependent and autocorrelated growth persist through time, they typically lead to larger growth depensation than within-individual variation in growth rates.

Because methods to separate the contributions of all three mechanisms acting simultaneously would be both complicated and data-hungry, we focus on the relative contribution to growth depensation of within- and among-individual variation in growth. We thus assume that individual growth rates are independent of size, or equivalently that a cohort's mean body size grows linearly through time. Although this assumption may seem restrictive, many organisms grow approximately linearly in size over some window in their ontogeny [33], [34]. More generally, our method will apply whenever body size data can be transformed to be a linear function of time. For example, if organisms grow exponentially with time (a common pattern early in ontogeny: [33]–[35]), then the solution is particularly easy: log-transforming the data automatically makes our method applicable. More generally, as long as we can fit a nonlinear growth curve to the data, we can invert the estimated growth curve and use it to linearize the data: we give an example of this approach in the case study below.

In the past, teasing apart the relative importance of within- and among-individual variation in growth for growth depensation has required scientists to mark individuals and follow each individual's growth pattern [3], [36], or to create distinct size classes in a starting cohort and monitor the intermixing of size classes [4]. In many ecological systems, neither of these approaches is feasible. Marking individuals can bias results by reducing survival and reproduction [37], [38], leading to ethical concerns [39]. Marking also requires extra time and effort that limits the scope of studies. There's a tradeoff between the quantity and detail of data that researchers can collect with a given amount of effort; this method makes it possible to use data containing less detail, but requires more of it. Lavine *et al.* (2002) [40] describe a method to estimate seedling mortality without marking individuals, using only observations of the numbers of old and new seedlings through time. Our method for quantifying among-individual variation is similar, fitting a model of the expected changes in variance through time to repeated measures of a cohort's variance.

## Data

### Simulated growth data

Our first “data” set is simulated growth data for a range of experimental designs (number of evenly spaced sampling times, number of individuals sampled across times) and growth parameters (increase in variance, 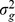, and strength of growth autocorrelation, *ρ* ^2^; all other parameters can be set to 1.0 without loss of generality). The increase in variance (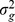) was set to 4, 16, 32, 64, 128, and 256; this range includes values observed in empirical studies on tadpoles [3]. The growth autocorrelation parameter *ρ* ranged from 0.1 to 0.9 by increments of 0.1. Observations were sampled at *n_t_* = 6, 8, 16, or 32 evenly spaced time steps for cohorts composed of 8, 16, 32, 64, 128, 256, and 512 individuals.

Growth autocorrelation was simulated by assigning each individual a normally distributed mean growth rate with mean *g* and variance 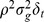 where *δ_t_* = 1/(*n_t_*−1). (Scaling the growth variance by *δ_t_* was done to make the total change in variance over the simulation independent of the number of time steps.) Within-individual variation was simulated for each individual at each time step by choosing random deviates with mean 0 and variance 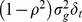. We simulated 1000 replicates for each combination of parameters in order to get precise estimates of power and coverage. For the purposes of testing our new method, we ignored individual ID, but we retained the information for the purposes of quantifying the power loss due to unidentifiability of individuals (*Repeated Measures* Section).

### Red-eyed tree frog data

We also analyzed data from an experiment designed to quantify density dependent growth for red-eyed treefrogs (*Agalychnis callidryas*) with either pulsed or gradual resource inputs (Appendix S1). The experiment was conducted between June and August 2008 at the Smithsonian Tropical Research Institute in Gamboa, Panama. The data include a total of 5609 individual body size measurements, spread across 6 time steps (every 5 days from hatching until 25 days old) in 10 tanks within 5 density treatments (5 to 100 individuals per tank) crossed with two resource levels (pulsed vs. gradual); the experiment was run in 4 replicate blocks. Body size was measured as total length, in millimeters.

### Ethics Statement

Permission to conduct this research in Panama was granted by Autoridad Nacional del Ambiente de Panamá (permiso no. SE/A-41-08) and the Smithsonian Tropical Research Institute (STRI). This research was conducted under Boston University Institutional Animal Care and Use Comittee (IACUC) protocol 08-011 and STRI's IACUC protocol 2008-04-06-24-08.

## Methods

We first derive the equations for the changes in cohort variance over time as a function of average growth rate, total variance in growth rate, and level of growth autocorrelation (non-technical readers can safely skip this section). We then discuss our protocol for simulating cohort growth dynamics to test the statistical power of our approach and summarize the practical aspects of the estimation procedure for researchers interested in applying the method to their own data. We compare our method to standard repeated measures methods that are available only when individuals are marked. Finally, we add size-dependent mortality to the data simulations and describe its effects on parameter estimates.

### Derivation

Suppose that individuals in a cohort grow linearly with mean growth rate *g* per time step 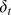. Each individual, with index *i*, consistently deviates from this average growth rate by 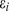, a normal deviate with mean 0 and variance 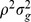. (Assuming normality is convenient for statistical inference on the parameters, but the derivation depends only on the mean and variance of this and other values.) At each time step, each individual's growth rate also has an uncorrelated deviation 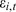 with mean 0 and variance 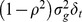. Then an individual's size changing through time can be modeled as
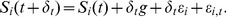
(1)Modeling an individual's growth in this way is equivalent to using two normal distributions with unrelated variances; parameterizing the model in terms of 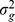 and *ρ* aids interpretation.

The cohort's size variance increases quadratically through time when *ρ* ^2^>0:
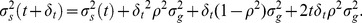
(2)
Appendix S2 gives a more detailed derivation.

Without loss of generality, we scale the units of *t* so that *t* ranges from 0 to 1 during the period of observation, so that 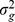 is the total increase in cohort variance during the period of observation; set average growth rate *g* (which does not appear in the variance equation (2)) to 1.0; and set the initial variance 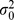 to 1.0, equivalent to setting the units of size – this also redefines 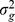 as the *relative* increase in variance over the observation period.

### Model Fitting

Because our model assumes only process and not measurement error, we fit the parameters by step-ahead prediction, equivalent for a normal response to fitting the between-step changes in variance as independent and normally distributed values with mean [41]

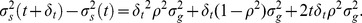
(3)Inspection of eq. 3 shows that the one-step change in variance is a linear function of *t*. As long as the maximum likelihood estimates (MLEs) of *ρ* and 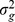 are within the interior of their feasible ranges (i.e., 0<
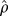
<1 and 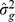
>0), we can fit a linear regression model for the change in variance as a function of time and use the estimated intercept and slope to solve for 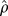 and 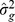. However, we still need to use nonlinear maximum likelihood estimation (1) in cases where the MLEs lie on the boundary (which is common when working with small, noisy data sets) and (2) in order to find reliable, likelihood profile confidence intervals for the parameters. We developed R code to compute starting values from linear regression as described above (constraining the starting values to lie on the boundaries of the feasible region where necessary) and using AD Model Builder [42], [43] or the bbmle package [44] in R to refine the estimates of the MLE where necessary and generate 95% likelihood profile confidence intervals on *ρ* (bbmle version 1.0.5.2, ADMB version 11.1, R2admb version 0.7.4, R version 2.15).

For our simulated data sets, we used the basic approach above and calculated statistical power (fraction of the time that the null hypothesis of *ρ* = 0 could be rejected based on the 95% confidence intervals) and coverage (proportion of simulations in which the 95% confidence intervals contained the true value of *ρ*).

Our red-eyed treefrog data showed clearly nonlinear (and decelerating) patterns of increasing size over time, We fitted a saturating-exponential model (size = *c*(1−exp(−(*b+dt*)) to the data aggregated at the level of tank averages, and used the linearizing transformation −log(1−*size*/*ĉ*) before applying the method above. The linearizing transformation fails when individuals have sizes greater than the estimated asymptotic size, *ĉ*; this was common for the higher-density treatments (75 and 100 individuals per tank), so we discarded these treatments completely, while discarding measurements larger than *ĉ* individually from the rest of the data set.

### Procedures

A step-by-step protocol for quantifying growth autocorrelation with our method is as follows: (1) Confirm that the mean growth rates of the cohort are roughly independent of the mean body size (or equivalently that growth is approximately linear), transforming the data (e.g. by taking logarithms, or fitting a growth model and applying the inverse growth-curve function as described above) if necessary. (2) Calculate the cohort's variance at each time step and take the differences to find the change in variance at each time step. (3) Estimate *ρ* from the data and the equation for change in size variation [3]. (4) Use likelihood profiling to find 95% confidence intervals for *ρ*. We have developed an R package, unmarkedAutocorrelation, that implements steps 2–4 (Appendix S3).

### Repeated Measures

When it is possible to mark individuals, more traditional repeated measures analyses can be used to estimate growth autocorrelation. To compare our method to repeated measures methods we fit a linear mixed model (LMM) to individual growth rates with a random effect of individual, using the same simulated data described above. We fit the model using lmer from the R package lme4 version 0.999999-0 [45]. We estimated *ρ* ^2^ from the variance of the random effect of individual and the residual variance of the fitted model:
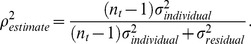
(4)To test if individual growth rates varied significantly (equivalent to testing the null hypothesis *ρ* = 0), we did an exact restricted likelihood ratio test on the random effect of individual using the function exactRLRT from the R package RLRsim version 2.0-10 [46].

### Size Dependent Mortality Simulations

Our model assumes that all individuals survive throughout the experiment. However, this assumption may be violated in experimental and especially in observational studies. The worst-case scenario is when individual mortality rates depend on size; we tested our method's performance in this scenario, specifically assuming that smaller individuals have a higher mortality rate (Appendix S4). Each individual survived according to a Bernoulli trial at each time step, with a probability equal to a logistic function of its size at time *t* scaled by the duration of the time step:
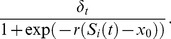
(5)We used *r* = 0.4 and *x_0_* = −10, −9, −8, and −7. For each value of *x_0_* and combination of parameters specified above, we estimated *ρ* for 1000 replicate simulations.

## Results and Discussion

### Simulation results

**Figure 2:**
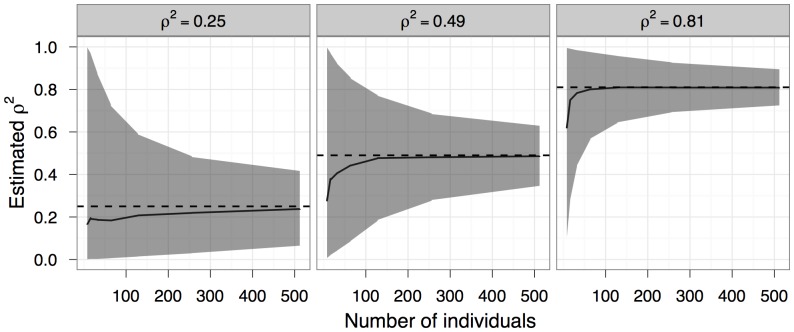
Estimates of growth autocorrelation *ρ* ^2^. Estimates of *ρ* ^2^ (solid lines), true values of *ρ* ^2^ (dashed horizontal lines) and 95% confidence intervals (gray ribbons), averaged over 1000 replicates for each parameter combination. Number of time points *n_t_* = 16.

Sampling more individuals improved point estimates and narrowed confidence intervals (Figure 2). Sampling more time points (in the range 6 to 32) had negligible effect on point estimates of *ρ*. Fewer time points gave slightly narrower (undercovering) confidence intervals (the confidence intervals for *ρ* ^2^ were 0.07 to 0.13 units narrower for 6 time points compared to 32 time points). In simulations with 6 or 8 time points, confidence intervals contained the true value of *ρ* slightly less than 95% of the time, but always gave above 90% coverage. When fewer than 50 individuals were sampled, 95% confidence intervals contained the true value of *ρ* more than 95% of the time (i.e. overcoverage). Thus, we recommend sampling a minimum of 50 individuals on more than 8 occasions. Increases in variation, 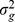, had no effect on bias or confidence interval width.

**Figure 3:**
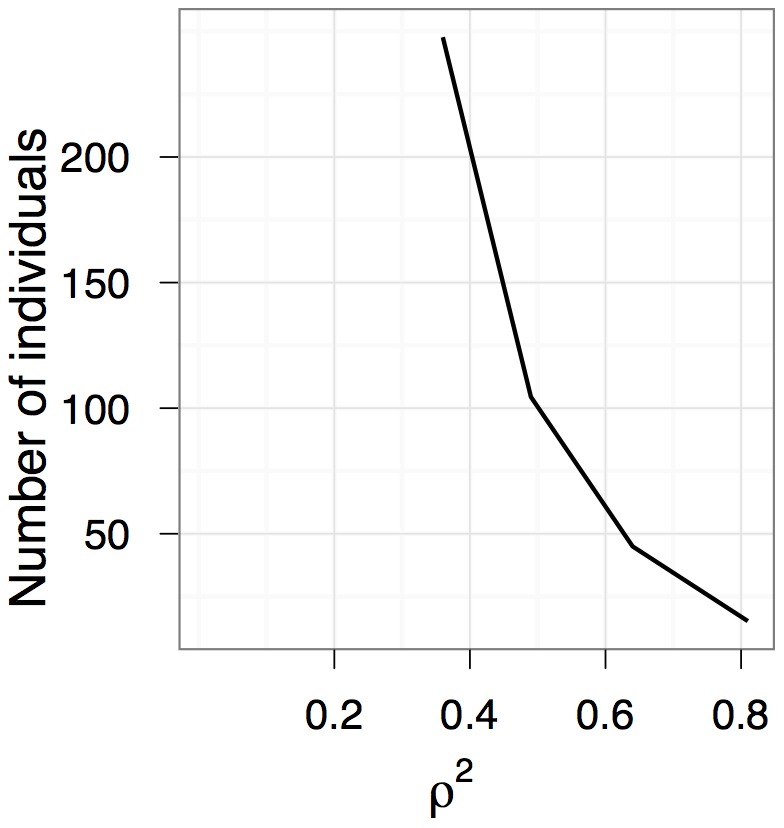
Number of individuals needed to detect positive growth autocorrelation. The line represents the minimum number of individuals in a cohort needed to statistically detect that *ρ* ^2^ is greater than 0 at least 80 percent of the time, based on the 95% confidence intervals of 1000 simulations with 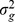 = 16 and *n_t_* = 16 (see *Simulating growth data* section for details). When *ρ* ^2^<0.36, more than 512 individuals are needed, beyond the simulated range.

Our simulation results can be used to guide experimental designs for detecting growth autocorrelation in cohorts of unmarked individuals. Preliminary growth data from pilot lab or field studies, or data from the literature, can be used to guess an approximate *ρ* ^2^. Given this information, researchers can use Figures 2 and 3 to make decisions about feasible precision and necessary sample sizes.

At a minimum, researchers will want to confirm whether observed growth depensation is the result of growth autocorrelation (i.e., to test the null hypothesis that *ρ* = 0 versus the alternative that *ρ*>0). The number of measured individuals needed for 80% power to detect *ρ* greater than zero depends strongly on the true value of *ρ* (Fig. 3). For example, at a true value of *ρ* ^2^ equal to 0.64, only 30 individuals are needed for 80% power (although with fewer than 50 individuals, estimates of *ρ* ^2^ may be biased: Fig. 2). For true values of *ρ* ^2^ = 0.36 and 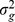 = 16, then 240 individuals are needed for 80% power. For the simulated experiments, only values of *ρ* ^2^ greater than 0.36 were ever distinguishable from zero with 80% power, regardless of sample size.

### Comparison of variance-pattern and repeated-measures approaches

**Figure 4:**
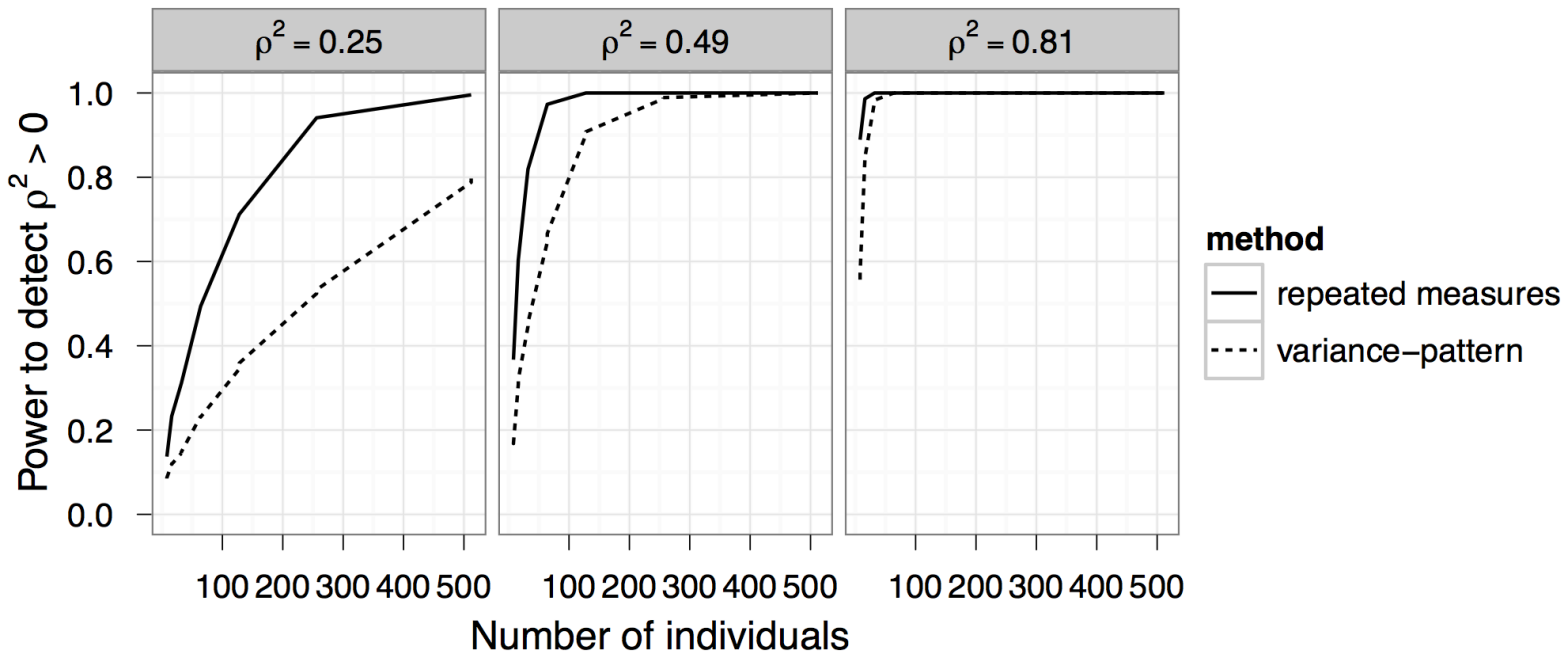
Power comparison with repeated-measures approaches. Our method for detecting growth autocorrelation (dotted line) is less powerful than exact restricted likelihood ratio tests on linear mixed models fit to data on marked individuals (solid line).

When individuals of the study species can be marked, traditional repeated-measures analyses can be used to estimate growth autocorrelation. As more individuals are sampled, both variance-pattern and repeated measures methods approach 100% power, although repeated measures power is always higher and increases more rapidly (Fig. 4). Nevertheless, variance-pattern power never has more than 40% less power in the range of scenarios we examined. Both methods are more powerful when detecting larger true values of *ρ* ^2^.

### Bias due to size-dependent mortality

In simulations incorporating size-dependent mortality, estimates of *ρ* were biased downwards. The strength of the bias increased with the proportion of individuals that died. Estimates of 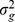 were also biased downward with increasing magnitude as a larger proportion of individuals died. Because smaller individuals were selectively removed, the cohort's variance increased by less than the nominal amount, 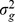. With less final variance, the cohort's change in variance through time followed a more linear, less quadratic pattern than predicted in the absence of mortality. Mortality rates were higher in simulations with larger values of 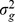, and hence bias increased, because the larger cohort variance resulted in more individuals falling within the high-mortality size range determined by equation 5. For a given value of *x_0_*, realized mortality varied greatly; realized mortality is a better predictor of bias, as well as being more directly related to ecological information that would be available to empirical researchers. Fits of *ρ* to simulated data with less than 5% mortality were biased by −.11 on average; 5–10% mortality caused average bias of −.17; 10–20% mortality caused average bias of −.27; 20–30% mortality caused average bias of −.37. For a figure of simulation results and simulation code with mortality, see the online supplement: Figure S1 and Appendix S4.

### Red-eyed treefrog case study

**Figure 5:**
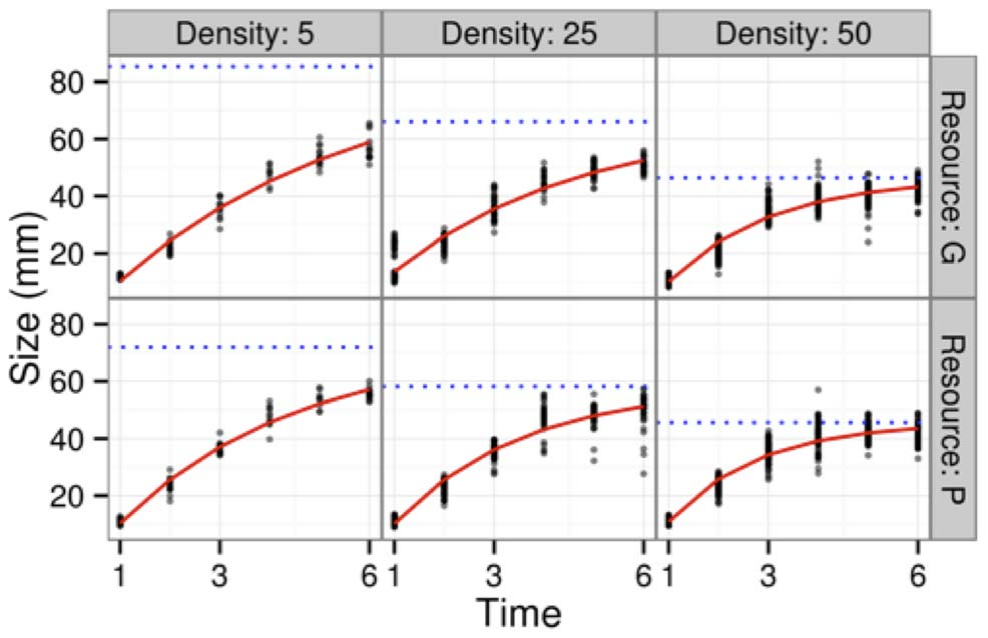
Data on red-eyed tree frogs: size (total length) in mm vs. time in days. Points represent tank means; red lines are estimated Michaelis-Menten growth curves; blue dotted lines are estimated asymptotic sizes.

**Figure 6:**
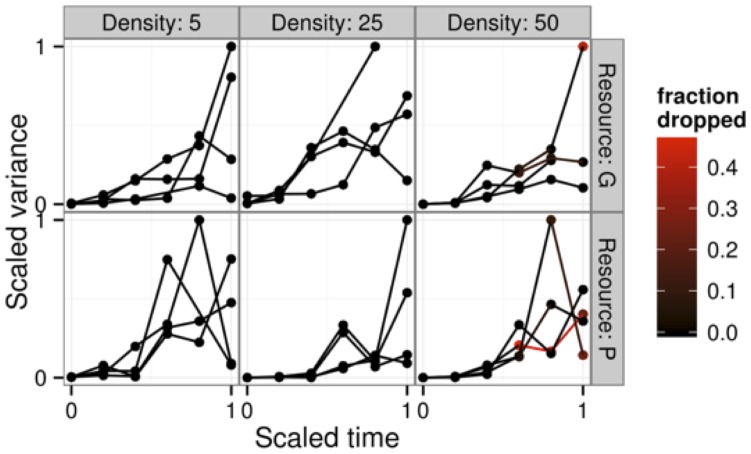
Estimated scaled variance per tank over time in different treatment combinations. Color scale represents the fraction of individual measurements dropped because they exceeded the estimated asymptotic size for the treatment combination (and hence could not be used in our linearizing transformation).

While we were successful in linearizing the growth curves for the red-eyed treefrog data (Figure 5 and Appendix S1), the data set was by and large too small to resolve information about growth autocorrelation (Figure 6). Given that the treefrog data consisted of 5—50 individuals measured at 6 time points, our power curve (Figure 3) suggests that we would only expect to detect autocorrelation at levels of *ρ* ^2^>0.7 at best. When we fitted the model at the level of individual tank replicates (i.e. for each of 4 block-tank combinations in each resource-density combination), we found the confidence regions generally spanned the entire range of autocorrelation. In three cases (one replicate each at densities of 5, 25, and 50 per tank in the good-resource treatment) the 95% lower bound was greater than 0 (*ρ* ^2^>0.35, 0.77, and 0.63 respectively), and in each of the cases the MLE was at *ρ* ^2^ = 1.0. When we pooled the data either to the level of the treatment (density∶resource combination), or to a single overall data set, we were unable to make any definitive statements about growth autocorrelation despite the larger effective sample size, probably due to the variation within and among treatments. In principle it would be possible to try to fit random effects models to try to squeeze slightly more information out of the data set without pooling, but with only 4 replicates per treatment we suspect the data set would still be insufficient to make any definitive conclusions.

## Conclusion

Previous methods for detecting autocorrelation in individual growth trajectories require the marking of individuals, which is logistically or ethically infeasible in many ecological systems. We have shown that, with a large enough sample, one can detect growth autocorrelation observationally by analyzing the patterns of increasing variance in body size over time. This new technique allows researchers to choose how to best allocate their effort: they can sample more individuals without marking them (a cheaper and faster design) or mark fewer individuals (a design that gains more information per individual).

Our method does have some limitations – it is reliable only where mortality is relatively low (<5% based on simulations in Appendix S4 and Figure S1) or size-independent, and applies when the growth trajectory of individuals is linear (or can be transformed to linearity) over the course of the study. While designed in the context of a closed population in a controlled laboratory study, it should be applicable to open populations as long as conditions are homogeneous across the super-population being sampled, and as long as individuals can be clearly identified as belonging to an even-aged (but not equally sized) cohort. Future extensions could allow for the influences of nonlinear growth and size-dependent mortality, although teasing these different effects apart may be challenging.

In circumstances where large numbers of organisms can easily be sampled and measured at repeated intervals through time, but the same individuals cannot be recovered or identified, our method should provide a reasonably powerful method for quantifying growth autocorrelation. Better quantification of the patterns and genesis of size variation will help improve management through better predictions of population dynamics as well as furthering ecologists' basic understanding of ecological systems.

## Supporting Information

Figure S1 **Each panel contains results of fitting the model to data sets with different amounts of growth autocorrelation (** ***ρ*** **^2^ = 0.25, 0.49, 0.81).** Realized mortality rate (the proportion of individuals that died by the end of the experiment) is plotted on the x-axis. Estimated values of *ρ* ^2^ for each simulation are plotted as grey dots. Red lines represent the true value of *ρ* ^2^. Blue lines summarize the simulations grouped by the total increase in size variation that would have been realized without mortality (*σ* ^2^). Smooth functions were fit with B-splines with five degrees of freedom.
(TIFF)

Appendix S1 **Case Study.** Using the R package provided in Appendix S4, we apply our method to a data set that requires linearization.
(PDF)

Appendix S2 **Model Derivation.** Here we present equations describing how a cohort's variance in body size will change through time based on our assumptions.
(DOCX)

Appendix S3 **R package.** This appendix contains an R package called unmarkedAutocorrelation. It can be used to simulate growth data, fit our model to data, and estimate parameter values and confidence intervals. To install the package, change your working directory to the location of the downloaded appendix and type install.packages(“Appendix S3 -unmarkedAutocorr_0.1.1.tar.gz”, repos = NULL, type = “source”).
(GZ) Click here for additional data file.

Appendix S4 **Mortality Code.** This appendix contains R code to simulate a cohort growing and experiencing size-dependent mortality. The simulations are repeated 1000 times for each combination over a range of parameter values that control mortality and growth rates. Estimated parameters and realized mortality are stored in an array.
(R) Click here for additional data file.
