# Supplementary figures and images for: A Method for Detecting Positive Growth Autocorrelation without Marking Individuals
Source: PLoS One. 2013 Oct 28;8(10):e76389. doi: 10.1371/journal.pone.0076389 (PMC3810375; doi:10.1371/journal.pone.0076389)

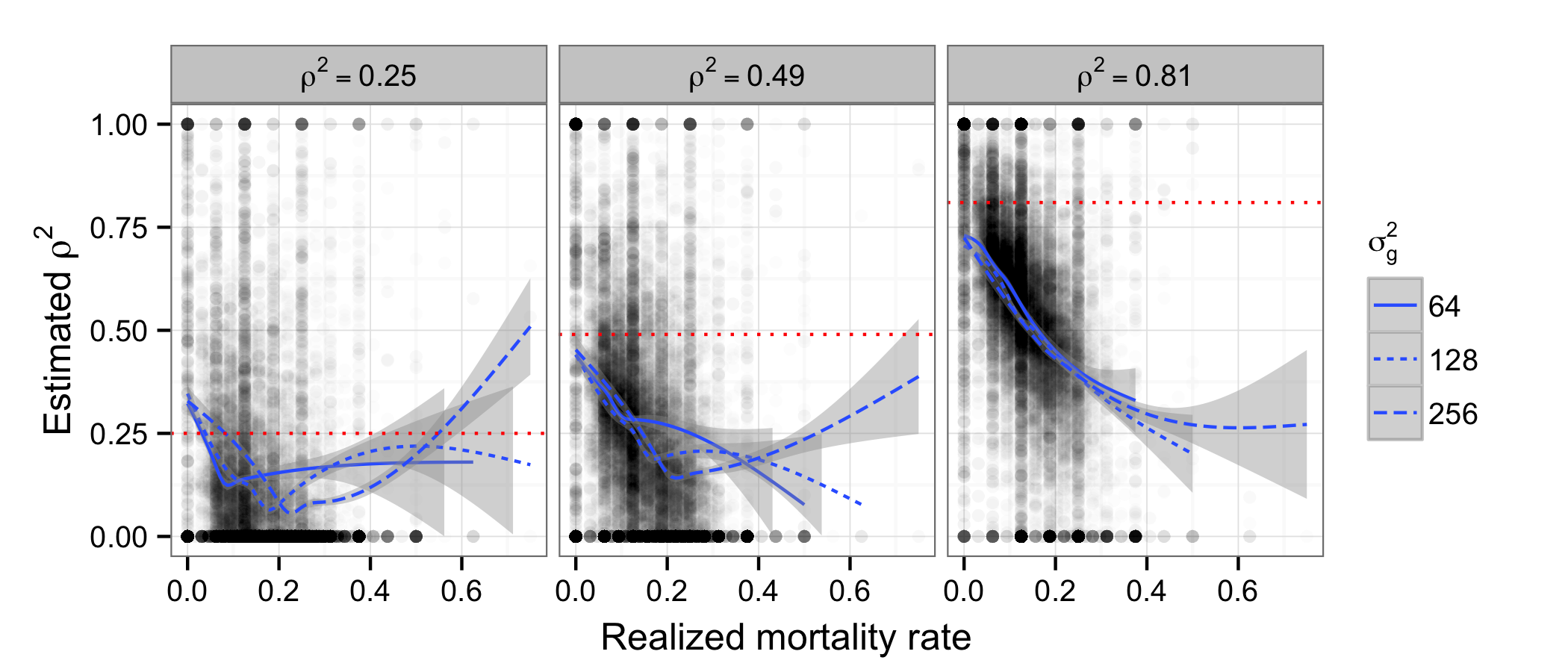

Supplement: Figure S1 — Each panel contains results of fitting the model to data sets with different amounts of growth autocorrelation ( ρ 2 = 0.25, 0.49, 0.81). Realized mortality rate (the proportion of individuals that died by the end of the experiment) is plotted on the x-axis. Estimated values of ρ 2 for each simulation are plotted as grey dots. Red lines represent the true value of ρ 2. Blue lines summarize the simulations grouped by the total increase in size variation that would have been realized without mortality (σ 2). Smooth functions were fit with B-splines with five degrees of freedom. (TIFF) [file pone.0076389.s001.tiff]
