# Supplementary material for: A Method for Detecting Positive Growth Autocorrelation without Marking Individuals
Source: PLoS One. 2013 Oct 28;8(10):e76389. doi: 10.1371/journal.pone.0076389 (PMC3810375; doi:10.1371/journal.pone.0076389)

Callidryas growth variance analysis

Ben Bolker and Mollie Brooks

March 5, 2013 @ 22:02

```
library("grid")      ## for unit()
library("plyr")      ## for ddply()
library("nlme")      ## for gnls(), nlsFit()
library("ggplot2")
zmargin <- theme(panel.margin=unit(0,"lines"))
theme_set(theme_bw())
library("gridExtra") ## for grid.arrange()
library("unmarkedAutocorr")
```

The data `dat_sum` (data summarized by tank) and `dat_ind` (individual-level data) are available when the `unmarkedAutocorr` package is loaded.

Further useful data transformations:

```
dat_sum2 <- na.omit(within(dat_sum,
               {
                 fBlock <- factor(Block)
                 fDensity <- factor(Density)
                 grp <- interaction(fDensity,Resource)
             }))
nlev <- with(dat_sum2,length(levels(fDensity))*length(levels(Resource)))
```

A first look (average size within each tank):

```
(g1 <- ggplot(dat_sum2,
              aes(x=Time.rep,y=avg))+geom_point()+
 facet_grid(Resource~Density)+zmargin+
 geom_line(aes(group=Block))+
 labs(x="Time",y="Average size (mm)"))
```

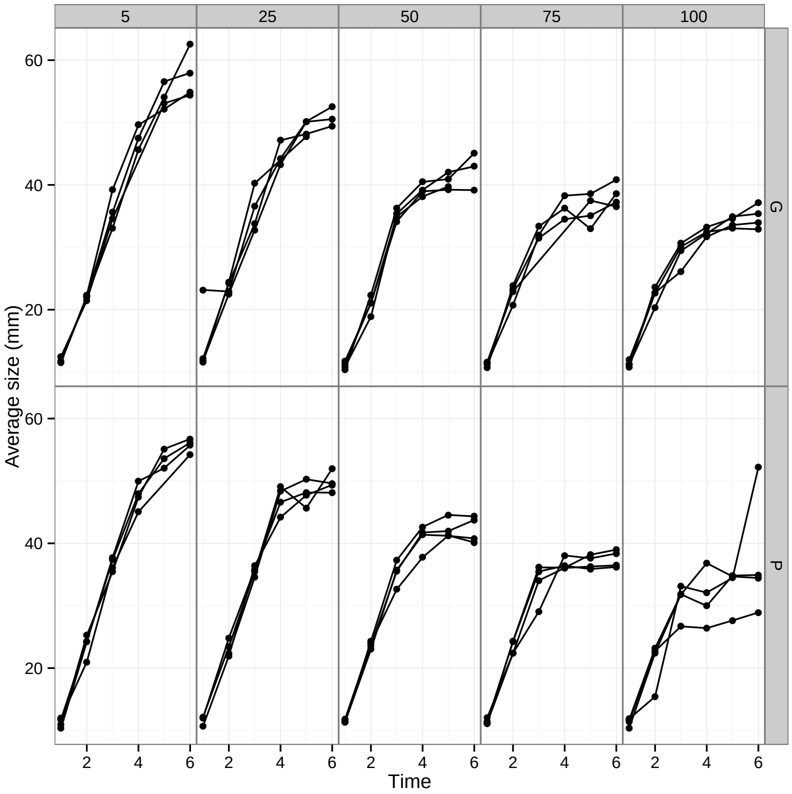

Two obvious choices for fitting this curve are monomolecular ($y \propto 1 - \exp(-x)$) and Michaelis-Menten ($y \propto x/(1+x)$). Both of these can be linearized, but there are issues in each case:

- Monomolecular: the linearizing transformation of $y = c(1-\exp(-(b+dx)))$ (this parameterization gives a maximum of $y(\infty) = c$ and a value of $y(0) = c(1-\exp(-b)))$ is $-\log(1-y/c)$. We have to estimate c from the data, and we get in trouble for any y value greater than c ...
- Michaelis-Menten: the standard linearizing transformation for $y = ax/(b+x)$ is $1/y = 1/a + b/x$ (in the old days this was called a Lineweaver-Burk plot; $x/y = (b+x)/a$ is another possibility (Scatchard plot). The problem with these is that they still don't make $f(y) = 1/y$ (1/size) a linear function of x (time), but of $1/x$ ...

We will use a monomolecular fit, coded as `SSasymp` in R. Baseline fit (pooled data):

```
nlsfit0 <- nls(avg~SSasymp(Time.rep,Asym,R0,lrc),
               data=dat_sum2)
```

We can use the results as starting values for `nlsList`, which gives the coefficients in the most convenient format:

```
nlsfit1 <- nlsList(avg~Asym+(R0-Asym)*exp(-exp(lrc)*Time.rep)|grp,
                   data=dat_sum2,
                   start=coef(nlsfit0))
```

`gnls` makes it more convenient to generate predictions:

```
nlsfit2 <- gnls(avg~SSasymp(Time.rep,Asym,R0,lrc),
                params=Asym+R0+lrc~fDensity*Resource,
                data=dat_sum2,
                start=rbind(coef(nlsfit0),
                     matrix(0,ncol=length(coef(nlsfit0)),
                     nrow=nlev-1)))
dat_sum2$pred <- predict(nlsfit2)
```

Construct a data frame containing the estimated asymptote for each treatment:

```
cc1 <- coef(nlsfit1)
maxval <- cc1[,"Asym"]
dmax <- with(dat_sum2,expand.grid(fDensity=levels(fDensity),Resource=levels(Resource)))
dmax <- transform(dmax,Density=as.numeric(as.character(fDensity)))
dmax <- cbind(dmax,cc1)
```

Plot the fits, and the asymptotes:

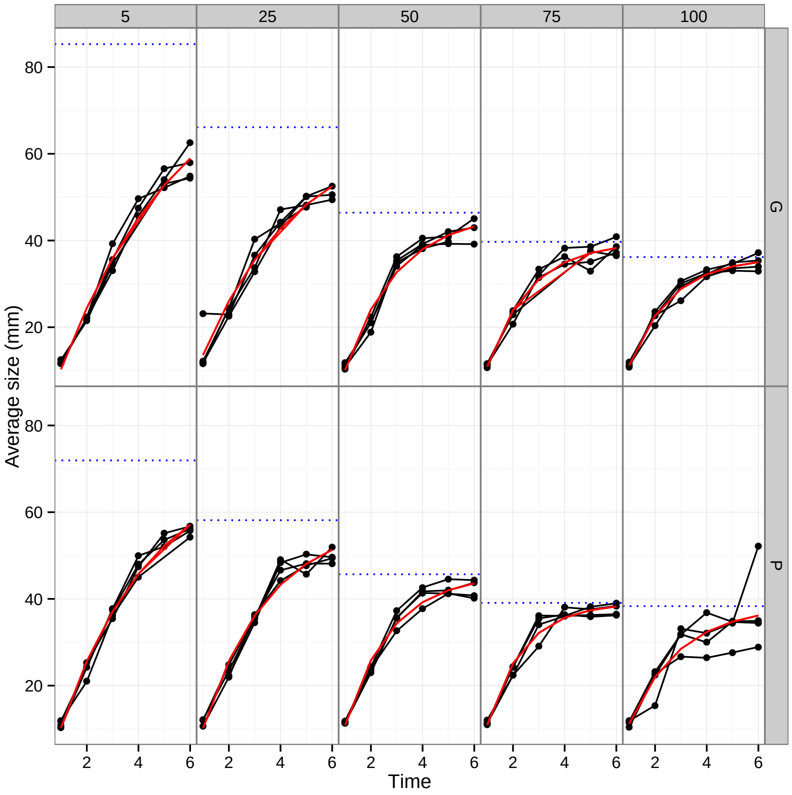

Looking at the same fits with the individual data:

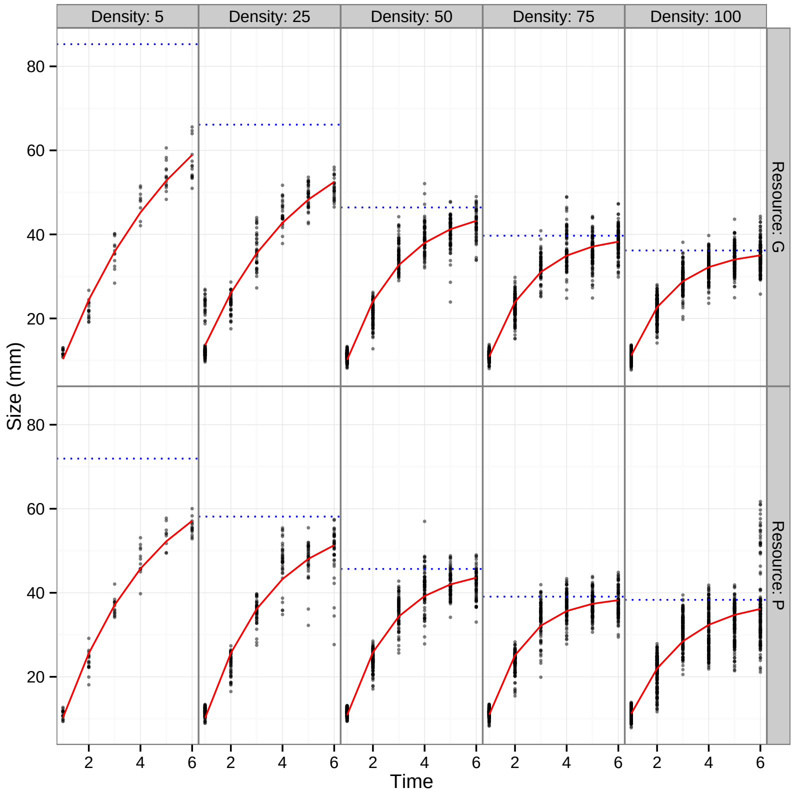

We will run into some trouble for cases where there are individuals larger than the estimated asymptote (especially densities 75 and 100): we will discard the higher densities, and keep an eye on the density=50 case as we proceed.

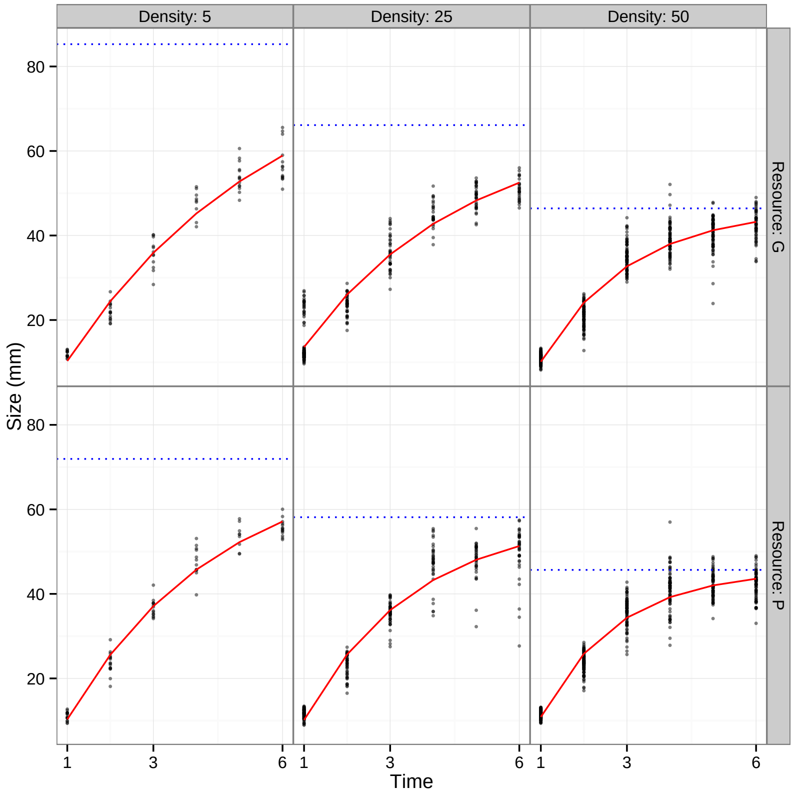

Now we will use the estimated curves to transform the individual-level data:

```
dat_ind2 <- droplevels(subset(dat_ind,Density<75))
dat_ind2 <- merge(subset(dat_ind2,select=-Date),
               subset(dmax,select=-fDensity))
dat_ind2$val_trans <- with(dat_ind2,-log((value-Asym)/(R0-Asym)))
```

We get a warning about `NaN` values: inspecting, we see that overall `100*mean(is.na(dat_ind2$val_trans))` = 2.2% of the cases are bad.

```
dat_ind2_agg <- ddply(dat_ind2,c("Time.rep","Block","Tank"),
                     function(x) {
                         with(x,
                              data.frame(avg_trans=mean(val_trans,na.rm=TRUE),
                                         sd_trans=sd(val_trans,na.rm=TRUE),
                                         n=nrow(x),
                                         frac_bad=mean(is.na(val_trans)),
                                         Density=Density[1],
                                         Resource=Resource[1]))
```

```
                    })
dat_ind2_agg <- ddply(dat_ind2_agg,c("Resource","Density"),
                      transform,
                      scsdvar=sd_trans^2/max(sd_trans^2))
```

```
g7 <- ggplot(dat_ind2_agg,
       aes(x=(Time.rep-1)/5,y=scsdvar,colour=frac_bad))+
    geom_point()+
    geom_line(aes(group=interaction(Block,Tank)))+
    facet_grid(Resource~Density,labeller=label_both)+zmargin+
    scale_colour_continuous(low="black",high="red",name="fraction\ndropped")+
    scale_x_continuous(name="Scaled time",breaks=c(0,1))+
    scale_y_continuous(name="Scaled variance",breaks=c(0,1))
ggsave("callidryas_variance.png",height=3,width=5,dpi=400)
```

So there's a lot of among-tank variance too.

```
fn <- "fitlist.RData"
if (!file.exists(fn)) {
    fitlist2 <- dlply(dat_ind2_agg,
                      c("Block","Tank"),
                      function(x) {
                          vfit(dvar=diff(x$sd_trans^2)) })
    rm(nt) ## hack
    fitlist3 <- dlply(dat_ind2_agg,
                      c("Block","Tank"),
                      function(x) {
                          vfit(dvar=diff(x$sd_trans^2),method="ADMB") })
    save("fitlist2","fitlist3",file=fn)
} else load(fn)
```

Put together tank-by-tank estimates and confidence intervals from both R and ADMB for comparison:

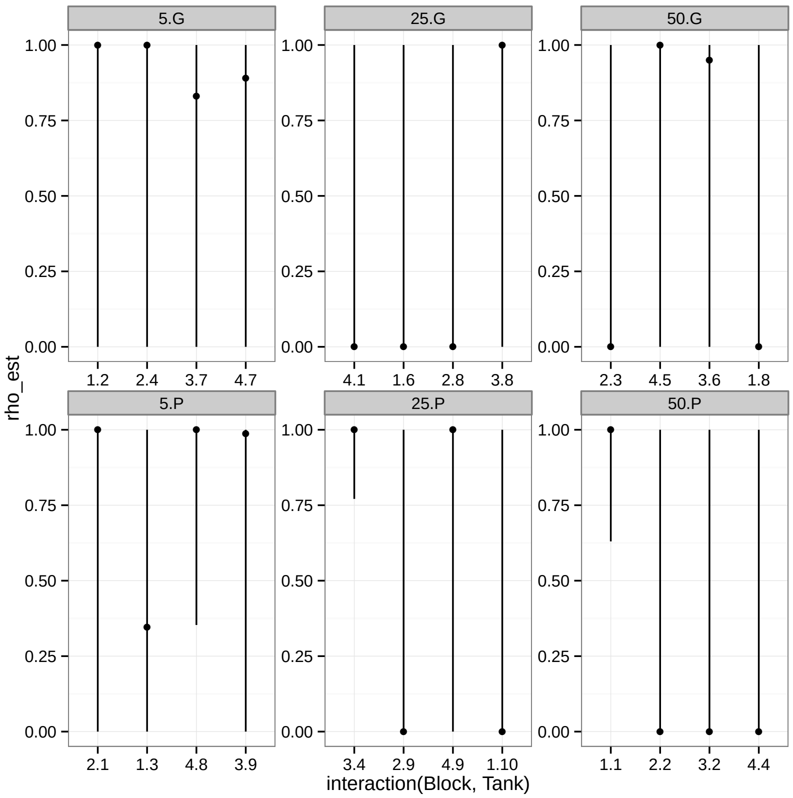

Which groups have positive lower bounds?

```
subset(mdat_results,method=="R" & rho_lwr>0)

##     method Block Tank Density Resource rho_est rho_lwr rho_upr  grp
## 71       R     4    8       5        P       1  0.3532       1  5.P
## 93       R     3    4      25        P       1  0.7710       1 25.P
## 114      R     1    1      50        P       1  0.6303       1 50.P
```

We're *nearly* done. The last (??) thing to do is to try to get a combined/pooled fit (random effects model would be nice but is a pain).

```
pooldat <- ddply(dat_ind2_agg,
            c("Block","Tank"),
            function(x) {
                with(x,data.frame(Block=Block[1],Tank=Tank[1],
                          tvec=head(Time.rep-1,-1)/6,
                          dvar=diff(sd_trans^2)))
            })
pooldat <- merge(pooldat,mdat_info)
```

```
fitlist4 <- dlply(pooldat,
                  "grp",
                  function(x) {
                      with(x,vfit(dvar=dvar,tvec=tvec,dt=1/6,nt=nrow(x)))
                  })
```

```
print(t(sapply(fitlist4,coef)),digits=3)

##            rho  var_g
## 5.G  1.00e+00 0.0383
## 25.G 1.89e-13 0.0390
## 50.G 9.93e-01 0.7897
## 5.P  9.78e-13 0.0234
## 25.P 1.00e+00 0.9586
## 50.P 3.33e-01 0.9847

t(sapply(fitlist4,function(x) x$ci))

##      2.5 % 97.5 %
## 5.G     NA     NA
## 25.G    NA     NA
## 50.G    NA     NA
## 5.P     NA     NA
## 25.P    NA     NA
## 50.P    NA     NA
```

After all that, if we lump everything together we get no detectable autocorrelation. Well, we can still fall back on the tank-by-tank results (which show a little bit). This is not really surprising given our power results. We have about 100 individuals per block/tank combo, 4 blocks/tanks per treatment, only 6 time steps — should we have expected to be on the edge anyway? (Consider also that the power for real, wonky data is likely to be less than the best-case scenario of simulated data.)

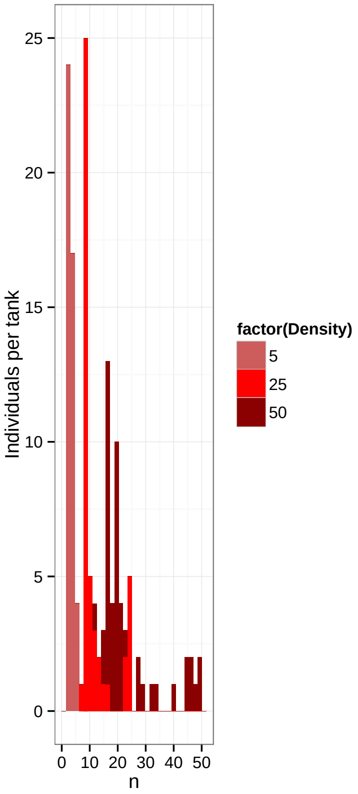

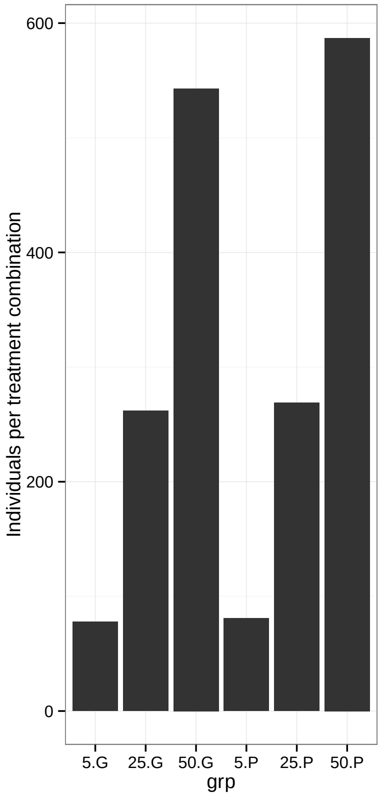

One last try: lump *all* of the data together.

```
(fit5 <- with(pooldat,vfit(dvar=dvar,tvec=tvec,dt=1/6,nt=nrow(pooldat))))

##
##  Growth autocorrelation fit (using R)
##
##
## Call:
## mle2(minuslogl = pfun, start = start1, method = "L-BFGS-B", data = list(dvar = dvar,
##     nt = nt, dt = dt, tvec = tvec), gr = pgfun, control = list(parscale = abs(unlist(star
##     trace = trace), lower = c(var_g = min_var_g, rho = 0), upper = c(var_g = Inf,
##     rho = 1))
##
## Coefficients:
##    rho  var_g
## 0.9116 0.4869
##
## Log-likelihood: 126.3
##
```

```
## Warning: optimization did not converge (code 52: ERROR: ABNORMAL_TERMINATION_IN_LNSRCH)
##
## Confidence intervals:
##  2.5 % 97.5 %
##     NA     NA
```

We still can't pick out confidence intervals from this.

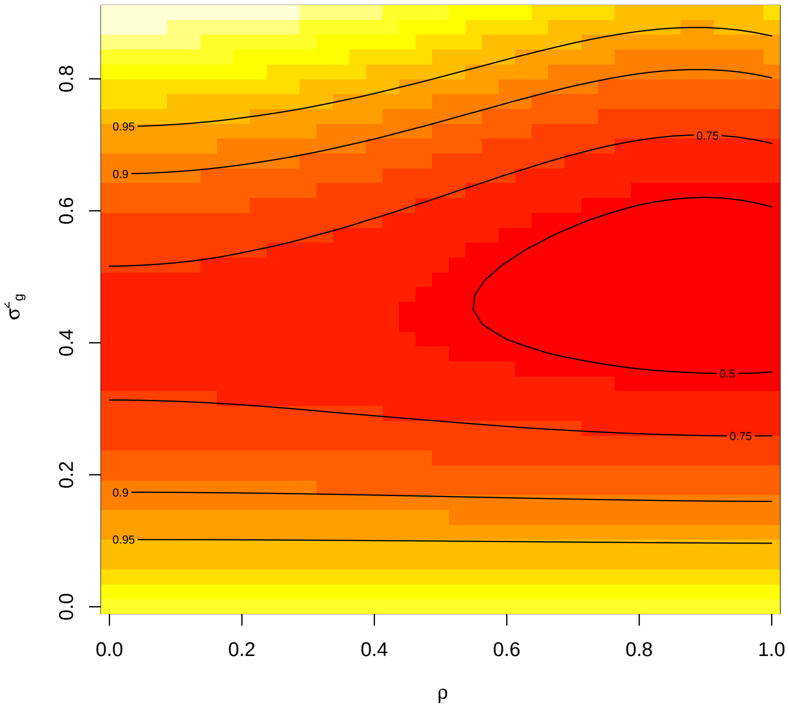

Supplement: Appendix S1 — Case Study. Using the R package provided in Appendix S4, we apply our method to a data set that requires linearization. (PDF) [file pone.0076389.s002.pdf]
