# Supplementary material for: A Method for Detecting Positive Growth Autocorrelation without Marking Individuals
Source: PLoS One. 2013 Oct 28;8(10):e76389. doi: 10.1371/journal.pone.0076389 (PMC3810375; doi:10.1371/journal.pone.0076389)
Supplement: Appendix S2 — Model Derivation. Here we present equations describing how a cohort's variance in body size will change through time based on our assumptions. (DOCX) [file pone.0076389.s003.docx]

**Appendix S2**

Assume individual *i*’s size through time follows:$S_{i}(t+\delta_{t})=S_{i}(t)+\delta_{t}g+\delta_{t}\varepsilon_{i}+\varepsilon_{i,t}$, where $\varepsilon_{i}$ has mean 0 and among-individual variance $\rho^{2}\sigma_{g}^{2}$, and $\varepsilon_{i,t}$ has mean 0 and variance through time$\left(1-\rho^{2}\right)\sigma_{g}^{2}\delta_{t}$. Let denote the expected value across individuals.

$$\begin{matrix} \sigma^{2}\left(t+\delta_{t}\right)=\left\langle\left(S_{i}\left(t+\delta_{t}\right)\right)^{2}\right\rangle-\left\langle S_{i}\left(t+\delta_{t}\right)\right\rangle^{2} \\ =\left\langle\left(S_{i}\left(t\right)+g\delta_{t}+\varepsilon_{i}\delta_{t}+\varepsilon_{i,t}\right)^{2}\right\rangle-\left(\left\langle S_{i}\left(t\right)\right\rangle+g\delta_{t}\right)^{2} \\ =\left\langle S_{i}\left(t\right)^{2}\right\rangle+2\left\langle S_{i}\left(t\right)\right\rangle g\delta_{t}+2\left\langle S_{i}\left(t\right)\varepsilon_{i}\delta_{t}\right\rangle+g^{2}\delta_{t}^{2}+\left\langle\left(\varepsilon_{i}\delta_{t}\right)^{2}\right\rangle+\left\langle\left(\varepsilon_{i,t}\right)^{2}\right\rangle-\left\langle S_{i}\left(t\right)\right\rangle^{2}-2\left\langle S_{i}\left(t\right)\right\rangle g\delta_{t}-g^{2}\delta_{t}^{2} \\ =\left\langle S_{i}\left(t\right)^{2}\right\rangle-\left\langle S_{i}\left(t\right)\right\rangle^{2}+2\delta_{t}\left\langle S_{i}\left(t\right)\varepsilon_{i}\right\rangle+\left\langle\left(\varepsilon_{i}\delta_{t}\right)^{2}\right\rangle+\left\langle\left(\varepsilon_{i,t}\right)^{2}\right\rangle\\ =\left\langle S_{i}\left(t\right)^{2}\right\rangle-\left\langle S_{i}\left(t\right)\right\rangle^{2}+2\delta_{t}\left\langle\left(S_{i}\left(0\right)+gt+\varepsilon_{i}t+\sum_{\tau=0}^{t} \varepsilon_{i,\tau}\right)\varepsilon_{i}\right\rangle+\left\langle\left(\varepsilon_{i}\delta_{t}\right)^{2}\right\rangle+\left\langle\left(\varepsilon_{i,t}\right)^{2}\right\rangle\\ =\left\langle S_{i}\left(t\right)^{2}\right\rangle-\left\langle S_{i}\left(t\right)\right\rangle^{2}+2\delta_{t}\left\langle\varepsilon_{i}^{2}t\right\rangle+\left\langle\left(\varepsilon_{i}\delta_{t}\right)^{2}\right\rangle+\left\langle\left(\varepsilon_{i,t}\right)^{2}\right\rangle\\ =\sigma^{2}\left(t\right)+2\delta_{t}\rho^{2}\sigma_{g}^{2}t+\delta_{t}^{2}\rho^{2}\sigma_{g}^{2}+(1-\rho^{2})\sigma_{g}^{2}\delta_{t} \end{matrix}$$
